# Monitoring droughts in Eswatini: A spatiotemporal variability analysis using the Standard Precipitation Index

**DOI:** 10.4102/jamba.v11i1.712

**Published:** 2019-10-24

**Authors:** Daniel H. Mlenga, Andries J. Jordaan

**Affiliations:** 1 Disaster Management Training and Education Centre for Africa, Faculty of Natural and Agricultural Sciences, University of the Free State, Bloemfontein, South Africa

**Keywords:** Standard Precipitation Index, drought, rainfall, spatial and temporal variability, Eswatini, drought monitoring

## Abstract

The spatiotemporal analysis of drought is of great importance to Eswatini as the country has been facing recurring droughts with negative impacts on agriculture, the environment and the economy. In 2016, the country experienced the most severe drought in over 35 years, resulting in food shortages, drying up of rivers as well as livestock deaths. The frequent occurrence of extreme drought events makes the use of drought indices essential for drought monitoring, early warning and planning. The aim of this study was to assess the applicability of the Standard Precipitation Index (SPI) for near real-time and retrospective drought monitoring in Eswatini. The 3-, 6- and 12-month SPI were computed to analyse the severity and onset of meteorological drought between 1986 and 2017. The results indicated that the climate of Eswatini exhibits geospatial and temporal variability. Droughts intensified in terms of frequency, severity and geospatial coverage, with the worst drought years being 1985–1986, 2005–2006 and 2015–2016 agricultural seasons. Moderate droughts were the most prevalent, while the frequency of severe and very severe droughts was low. Most parts of the country were vulnerable to mild and moderate agricultural droughts. Spatial analysis showed that the most severe and extreme droughts were mostly experienced in the Lowveld and Middleveld agro-ecological zones. The 3-, 6- and 12-month SPI computations conducted in January detected the onset of early season drought, thereby affirming the applicability of the index for monitoring near real-time and retrospective droughts in Eswatini. Drought monitoring using the SPI provides information for early warning, particularly in drought-prone areas, by depicting a drought before the effects are felt.

## Introduction

Drought is a pressing economic, social and environmental issue that is of great importance to Eswatini, similar to the rest of southern Africa, where for the past few decades the region has been affected by recurring droughts which had negative impacts on rain-fed agriculture, the environment, the economy and the livelihoods of people. The increased frequency in drought occurrence has triggered an increased scientific and social interest; this is in relation to future climatic conditions in the region, especially where modelling experts have predicted that drought years will be more common and severe in southern Africa and the impacts more significant (IPCC 2012, 2013). To mitigate, therefore, the impending challenges, the key is to understand drought and its natural and social dimensions so as to enhance drought risk management. This is in an effort to increase society’s coping capacity, which will in turn lead to greater resilience and a reduced need for government or donor interventions in the form of disaster assistance.

Drought is spatially variant and context dependent, thereby making it difficult to describe accurately. Although the effects and impacts of drought events are well documented, a standardised method for monitoring drought conditions and quantifying the severity of drought does not exist. The drought index, however, is the most common tool used for monitoring drought conditions. A drought index can be used to quantify the moisture condition of a region, thereby detecting the onset and measuring the severity of drought events. Drought indices can be useful tools for providing information to decision-makers to predict crop yield (Kumar & Panu 1997), provide early drought warning information (Lohani & Loganathan 1997; Lohani, Loganathan & Mostaghimi 1998), calculate the probability of drought termination (Karl, Quinlan & Ezell 1987), determine drought assistance (Wilhite, Rosenberg & Glantz 1986) and make comparisons between different regions (Alley 1984, 1985; Dai, Trenberth & Karl 1998; Kumar & Panu 1997; Nkemdirim & Weber 1999; Soulé 1992).

Precipitation-based drought indices are applied to characterise drought conditions (Tadesse et al. 2008). The challenge, however, is the absence of continuous spatial rainfall data coverage, thereby reducing the ability to monitor and characterise detailed spatial and temporal patterns of drought (Manyatsi, Ntobeko Zwane & Dlamini 2015). The lack of agreed drought indicators and thresholds above or below by which a drought can be declared makes the objective of drought monitoring and drought declaration difficult and often late. This often results in loss of life and livestock, food insecurity and a significant financial impact on the economy. The aim of this study was therefore to assess the applicability of the Standard Precipitation Index (SPI) for near real-time and retrospective drought monitoring in Eswatini. The efficient use of the SPI and Normalized Difference Vegetation Index (NDVI) can improve drought monitoring and early warning in Eswatini.

### Standard Precipitation Index

Over the years, many drought indices were developed and used by meteorologists and climatologists around the world. The most commonly used index worldwide though is the SPI. McKee developed the SPI during the early 1990s (McKee, Doesken & Kleist 1993). The World Meteorological Organization (WMO) in 2009 recommended the SPI as the main meteorological drought index that countries should use to monitor and follow drought conditions (Hayes et al. 2011). Currently, many scientists prefer the SPI as an index for drought risk (Giddings et al. 2005; Gutman 1999; Hayes et al. 1999; Jordaan 2011). The index is recommended because it allows the comparison between different climates and locations. It can be used to analyse drought or anomalously wet periods at a particular timescale for any location in the world with daily precipitation records (McKee 1995; Moreira et al. 2008). Using the SPI as an indicator for drought monitoring, early warning drought disaster declaration will limit the arbitrary decision-making of politicians with scientifically based criteria.

It was designed to quantify the precipitation deficit for multiple timescales (McKee 1995). These timescales, days, weeks, months and years reflect the impact of drought on the availability of the different water resources. The SPI can be calculated for any location that has long-term precipitation data. The index can identify various drought types: hydrological, agricultural or environmental. The SPI is commonly calculated using 1-month, 3-month, 6-month, 9-month, 12-month and 24-month intervals. These timescales are appropriate for monitoring different types of drought and correspond to different drought impacts. The SPI calculation for any location is based on the long-term precipitation record for a desired period. This long-term record is fitted to a probability distribution, which is then transformed into a normal distribution so that the mean SPI for the location and desired period is zero (Belayneh & Adamowski 2012; Edwards et al. 1997).

For an in-depth comprehension of the meaning of SPI, an understanding of the concepts related to the SPI is essential. Mckee et al. (1993) and Jordaan (2011) reviewed and defined the SPI-related concepts as follows:

- *Accumulated precipitation* – This is the total rainfall during specified period.
- *Accumulated precipitation departure* – This is the amount by which the indicated accumulated precipitation is above or below the long-term average for exactly the same set of months.
- *Accumulated precipitation percent of average* – The accumulated precipitation, over the timescale of interest and extending through the end of the last month indicated, divided by the long-term average precipitation, which would be expected to accumulate over the same set of months, and then multiplied by 100.
- *Percentile or probability of nonexceedance* – This is the magnitude observed and regarded as the degree of ‘unusualness’.
- *Timescale* – The number of months extending through to the end of the current month.

McKee et al. (1993) used the classification system (Table 1) to define drought intensities resulting from the SPI. They also defined the criteria for a drought event for any of the timescales. A drought event occurs any time the SPI is continuously negative and reaches an intensity of −1.0 or less, and drought intensity can be determined by calculating the SPI values for all months within a drought event (McKee 1995; McKee et al. 1993). The event ends when the SPI becomes positive. Positive SPI values indicate greater than median precipitation and negative values indicate less than median precipitation.

**TABLE 1:** Drought classification based on Standard Precipitation Index.

SPI values	Class
≥ 2	Extremely wet
1.5–1.99	Very wet
1.0–1.49	Moderately wet
−0.99 to 0.99	Near normal
−1 to −1.49	Moderately dry
−1.5 to −1.99	Very dry
≤ 2	Extremely dry

*Source*: McKee, T.B., Doesken, N.J. & Kleist, J., 1993, ‘The relationship of drought frequency and duration to timescales’, in *Proceedings of the 8th conference on applied climatology*, vol. 17(22), pp. 179–183, American Meteorological Society, Boston, MA.

SPI, Standard Precipitation Index.

Each drought event, therefore, has a duration defined by its beginning and end, and intensity for each month that the event continues. The positive sum of the SPI for all the months within a drought event can be termed the drought’s ‘magnitude’ (WMO 2012).

## Applied methodology

### Study area

Eswatini is a landlocked nation almost entirely contained within the northeast corner of South Africa and located at the transition of the South African Plateau (reaching over 1500 m) to the Mozambican coastal plain. The country has a total area of 17 364 km^2^ and has approximately 12 220 km^2^ of agricultural land, or 71% of the total land area (FAO 2013). The country has four administrative districts (Hhohho, Manzini, Lubombo and Shiselweni) and is classified into four agro-ecological zones (AEZ), taking into account elevation, landforms, geology, soils, climate and vegetation: Highveld, Middleveld, Lowveld and Lubombo range.

The rainy season is from mid-October to mid-April, and the dry season is from mid-April to mid-October. Mean annual rainfall ranges from about 700 mm to 1500 mm in the northern Highveld (Table 2) to 200 mm in the southern Lowveld. The Middleveld annual rainfall ranges from 500 mm to 800 mm. The national long-term average rainfall is 788 mm per year. These large ranges indicate the fluctuating nature of Eswatini’s climate. These climatic conditions make the country very vulnerable to meteorological hazards such as drought, floods and gusty winds as well as lightening and epidemics during the wet and hot season. Mean annual temperature varies from 17 °C in the Highveld to 22 °C in the Lowveld (Government of Swaziland 2013).

**TABLE 2:** Rainfall in the agro-ecological zones of Eswatini.

Agro-ecological zone	Average rainfall
Highveld	700–1550
Middleveld	550–850
Lowveld	200–550
Lubombo Plateau	550–850

*Source*: FAO AQUASTAT Survey, 2005, *Irrigation in Africa in figures*, viewed 08 December 2016, from http://www.fao.org/ag/aquastat.

### Data set

**FIGURE 1:**
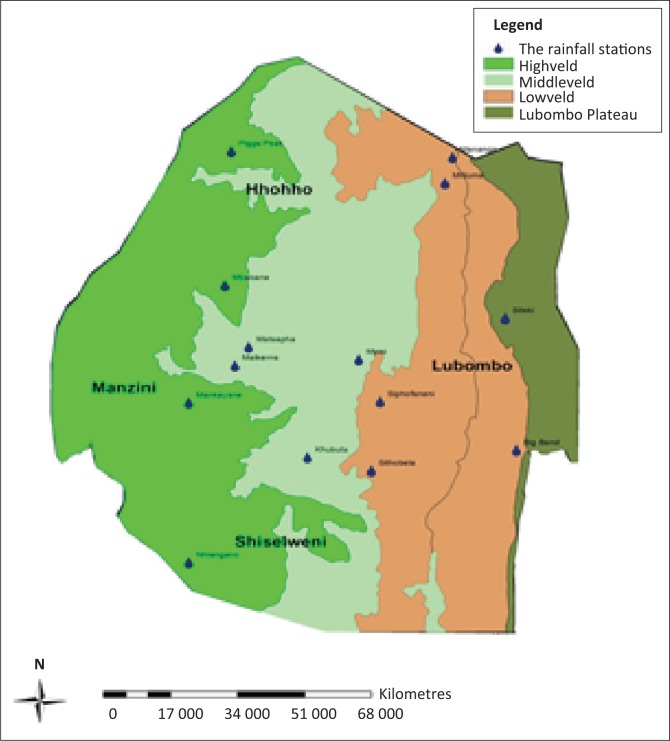
Map of Eswatini with agro-ecological zonation and the rainfall stations.

The SPI was used for drought monitoring for the time series from the period 1986 to 2017. Representative meteorological stations of the Eswatini Meteorological Service were selected with good data. The stations covered all agro-ecological regions (Figure 1) and administrative regions in Eswatini as presented in Table 3. Monthly rainfall data set was supplied by the Eswatini Meteorological Services. Only stations with full data were considered for analysis. To be able to present natural drought conditions, raw precipitation data were used. All the chosen precipitation stations displayed good data quality with no data gaps in the time series. This is because only rainfall stations that had the complete 32-year data set were selected. There was therefore no data filling or corrective homogeneity enforced.

**TABLE 3:** Meteorological stations and their geographic coordinates.

Agro-ecological region	Station name	Latitude (S)	Longitude (E)	Time series
Highveld	Mbabane	−26.33	31.15	1986–2017
	Nhlangano	−27.12	31.20	1986–2017
	Mankayane	−26.67	31.05	1986–2017
	Mhlume	−26.03	31.15	1986–2017
Middleveld	Matsapha	−26.53	31.30	1986–2017
	Piggs Peak	−25.82	31.42	1986–2017
	Khubutha	−26.83	31.47	1986–2017
	Mpisi	−26.43	31.53	1986–2017
	Malkerns	−26.55	31.87	1986–2017
Lowveld	Big Bend	−26.85	31.87	1986–2017
	Sithobelweni	−26.88	31.62	1986–2017
	Mananga	−26.00	31.75	1986–2017
	Siphofaneni	−26.67	31.68	1986–2017

*Source*: Eswatini Meteorological Service.

S, south; E, east.

### Computation of Standard Precipitation Index

The SPI was calculated according to the methodology explained by Giddings et al. (2005); however, the actual SPI computation was achieved through the use of DrinC software. The selection for software was based on its simplicity, such that it can be easily adopted for use in Eswatini. DrinC is a user-friendly tool software package that was developed for providing a simple, thorough adaptable interface for the calculation of several drought indices (Tigkas, Vangelis & Tsakiris 2015). The software operates on Windows platform and is programmed in Visual Basic.

The Eswatini rainfall data set of 2006–2017 was uploaded onto the DrinC software for manipulation. The SPI was calculated at 3-, 6- and 12-month timescales. The primary reference base in DrinC is the hydrological year (October to September); however, the study defined the hydrological year based on the Eswatini rainfall calendar. For the 3-month SPI, the hydrological year covered October, November and December. Ji and Peters (2003) found that the 3-month SPI is the most effective for monitoring drought impact on vegetation, especially when the 3-month period coincided with the peak growing season. The 6-month SPI hydrological year covered July to December, whereas the 12-month SPI hydrological year covered January to December. The 3-month SPI indicates the conditions of short-term drought, mostly soil moisture and drought stress with an impact on agriculture, while the 6- and 12-month SPIs indicate medium- to long-term droughts which affect ground water supplies and pasture conditions. The study therefore mapped drought severity at 3-, 6- and 12-month timescales in the four agro-ecological regions of Eswatini. The month of December was chosen for calculating the SPI for 3-, 6- and 12-month timescale as October is when the rainfall or agricultural season starts, whereas December is mid-season where the main cereal crops (maize) will be flowering. The 3-month period of October to December is therefore normally the critical wet season and therefore the onset of drought in this period will affect crop production.

Spatial representation of the SPI was performed using ArcGIS 10.1 where the geostatistical method of kriging was chosen for the representation of spatial distribution and intensity of the drought for the selected drought years. The SPI for the selected drought years, 1985–1986, 2004–2005, 2005–2006 and 2015–2016, was krigged to allow spatial interpolation of drought across AEZ.

### Ethical considerations

All authors have been personally and actively involved in substantive work leading to the manuscript, and will hold themselves jointly and individually responsible for its content. This article followed all ethical standards for research without direct contact with human or animal subjects.

## Results and discussion

### Precipitation over time

**FIGURE 2:**
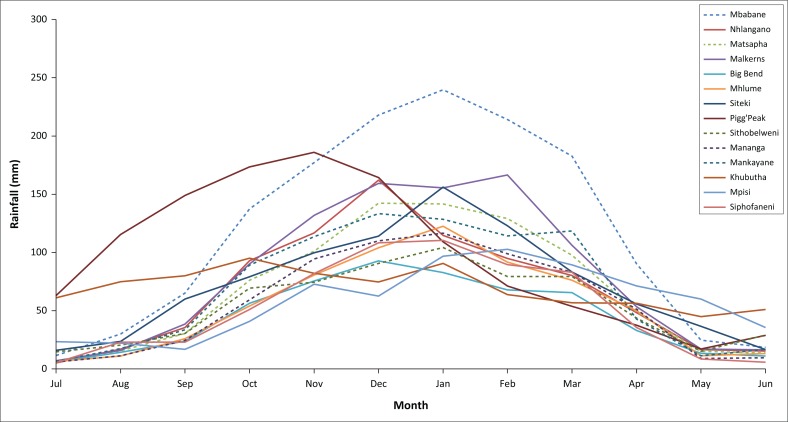
Mean historical monthly rainfall for Eswatini during the time period 1986–2017.

Precipitation level is an important factor affecting crop selection and ecological changes in a region. Precipitation over time graphs were made for all 14 stations in order to visualise the data time series of the precipitation values. Figure 2 presents the time series of Eswatini’s annual average precipitation between 1986 and 2017. The analysis of the precipitation over time is important for improved understanding of hydrometeorological processes and their long-term variations. This is because meteorological drought is considered a consequence of the negative deviation of rainfall from the mean and a most common indicator for drought (Wilhelmi & Wilhite 2002; Wilhite, Sivakumar & Wood 2000; WMO 2006). Cumulative rainfall calculations can be very useful with the prediction of dry periods and drought. The seasonal and the station-based rainfall patterns are also important for drought hazard assessment.

Figure 2 shows the mean historical monthly rainfall for 14 selected rainfall stations during the time period 1986–2017. The rainfall pattern between all the assessed rainfall stations was similar with the months of May and June being the dry winter months, where the rainfall was only received mostly in the Highveld with little or no rainfall in the Lowveld AEZ. The rainy season, which saw the progressive increase in the amount of rainfall received, started in September with the peak rainfall months in December and January. December and January coincide with summer crops’ main vegetative development and reproductive stages. Prolonged rainfall stress in these months will result in reduced crop yields. Analysing the time series of Eswatini’s annual average precipitation from 1986 to 2017, it was clear that there were years where rainfall was below the national average. The notable years were 1986, 1990, 1992, 1994, 2002–2003, 2005, 2007, 2008, 2011, 2014 and 2015–2016. The annual rainfall during the 32 years ranged from 363 mm in 2016 to 1309 mm in 2000, with an average of 819 mm. The lowest rainfall corresponds with the 2015–2016 El Nino, which was classified as one of the worst to occur in 50 years (Phys.org 2016).

**FIGURE 3:**
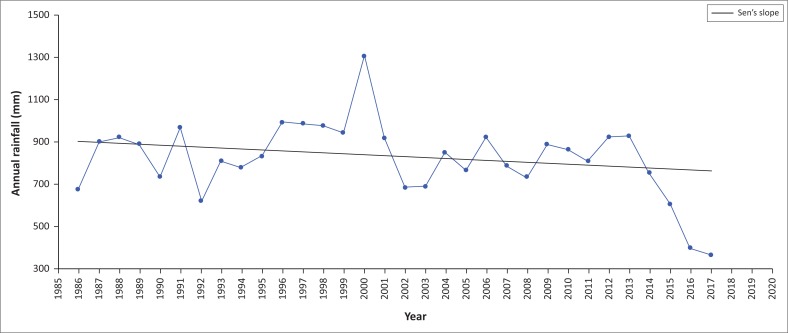
Annual rainfall trend for Eswatini (1986–2017).

Trend analysis was also performed on an annual scale to examine whether any trends existed in the data. The annual rainfall time series, averaged over the whole data set, is illustrated in Figure 3 with the corresponding Sen’s slope plotted. The variability around the mean (819 mm) was evident and pronounced, indicating a decrease over time in annual rainfall. The standard deviation of the annual rainfall shows higher values than the average, indicating the deviation from normal is considerable. Further, Mann–Kendal test results (Table 4) indicate a decreasing trend in annual rainfall across all meteorological stations. The Sen’s slope estimate of rate of decrease is −80 mm per year. However, the decreasing rate is not statistically significant at α = 0.05. The rainfall trends demonstrated a variation in the spatial variations of the Highveld, Middleveld and the Lubombo Plateau agro-ecological regions. The Lubombo Plateau had the highest mean rainfall, whereas the Lowveld had the lowest mean rainfall over the study period.

**TABLE 4:** Mann–Kendall trend test and two-tailed test (annual precipitation).

Statistic	Value
Kendall’s tau	−0.161
S	−80 000
Var(S)	3 802 667
*p*-value (two-tailed)	0.195
Alpha	0.05
Mean	819 mm
Standard deviation	179

Note: Trend equation: *y* −6.3389x + 13506; *R*² = 0.1103.

S, (Kendal score) indicates the slope of the trend (positive = upward, negative = downward).

### Drought severity temporal dynamics based on Standard Precipitation Index

**FIGURE 4:**
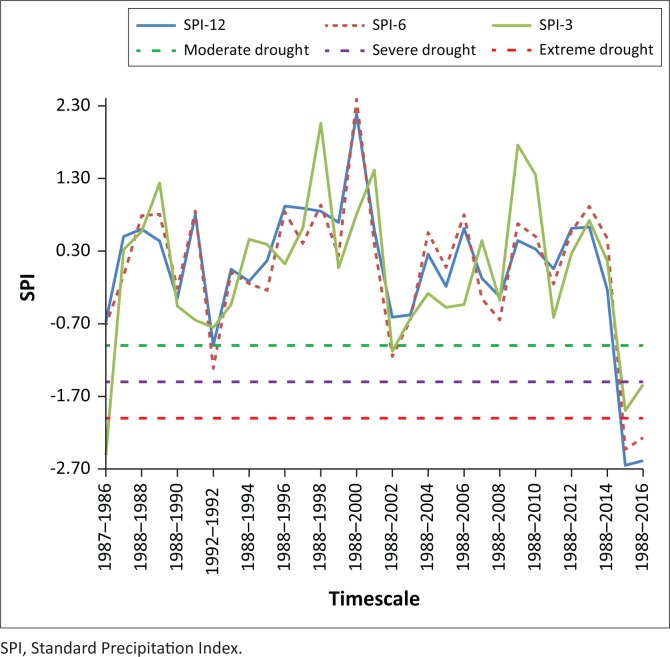
Standard Precipitation Index values of Eswatini for three different timescales (3, 6 and 12 months).

**FIGURE 5:**
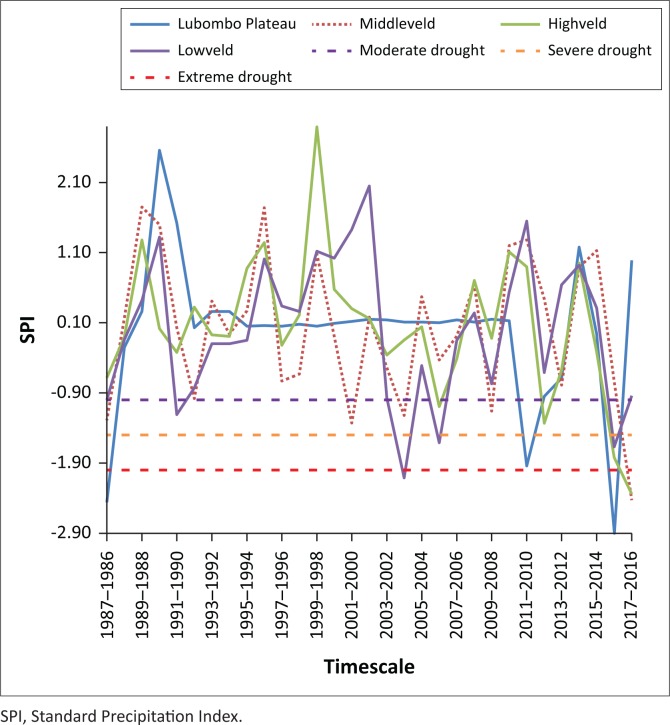
Three-month Standard Precipitation Index values for the Highveld, Middleveld, Lowveld and Lubombo Plateau agro-ecological zones.

The study calculated for the time series 1986 to 2017 using the SPI on 3-, 6- and 12-month timescales corresponds to the past 3, 6, and 12 months of observed precipitation totals, respectively. These timescales reflect the soil moisture conditions (SPI-3) or the underground waters, river flows and lake water levels (SPI-12) (Bokal 2006; Livada & Assimakopoulos 2007; Rouault & Richard 2003). Overall, the temporal SPI analysis using McKee et al. (1993), the SPI classification (Table 1) suggests that Eswatini experienced moderate to extreme drought episodes in the years 1986, 1990, 1992, 2006, 2012, 2014 and 2016. Moderate drought occurred in 1986, 1990 and 2012 (Figure 4), whereas severe drought occurred 1992 and 2006. The only extreme drought recorded was in 2016, which corresponds to the strongest El Nino period in over half a decade and the government drought disaster declaration in 2016 (NDMA 2016; Phys.org 2016; Swaziland National Vulnerability Assessment Committee 2016). The extreme drought result is consistent with the research by Rouault and Richard (2003), who highlighted that an SPI of −2.00 happens twice per century, from −1.50 to −1.99 about 4 times per century, and from −1.0 to −1.5 about 9 times per century. The minimum SPI value (−2.95) detected in 2016 across Eswatini was persistent, with varying severity in the different AEZs (Figure 5).

Differences were observed in the SPI results across different timescales. Moderate droughts were the most frequent within 3-, 6-, and 9-month categories, with the Middleveld having the highest occurrences. In 1992, for example, the 6-month and 12-month SPI indicated moderate drought, whereas the 3-month SPI showed no drought conditions. In 2006, the 3-month SPI indicated severe drought conditions, whereas the 6- and 12-month SPI indicated mild- to normal-drought periods. The 3-month SPI may be misleading, especially in areas where it is normally dry during that 3-month period. The differences in drought conditions may therefore be related, with the response of the short-term soil moisture conditions to precipitation during that short timescale for both 3-month and 12-month SPI (Saada & Abu-Romman 2017; WMO 2012). In 2006, it is evident that a drought event (as indicated by the 3-month SPI) occurred in the middle of a longer term drought, as evidenced by the 12-month timescale result. Therefore, it is important to compare the 3-month SPI with longer timescales (NDMC 2006; Rouault & Richard 2003).

Comparing the 3-month SPI across AEZs, most drought events were experienced in the Middleveld and Lowveld zones. Extreme droughts were experienced in all AEZs in 1986 and 2016. When the 3-month SPI was calculated for the different AEZs, there were parallels with the drought periods that were declared and documented in the EM-DAT database (EM-DAT 2016). This means that within the country there are spatial differences for drought severity and intensity. For moderate drought, however, the SPI calculation typifies the droughts, which were also declared in most of the AEZs. Therefore, the SPI can be used to typify drought by AEZ, with the Lowveld being the region that is most prone to drought.

### Drought severity spatial dynamics based on Standard Precipitation Index

**FIGURE 6:**
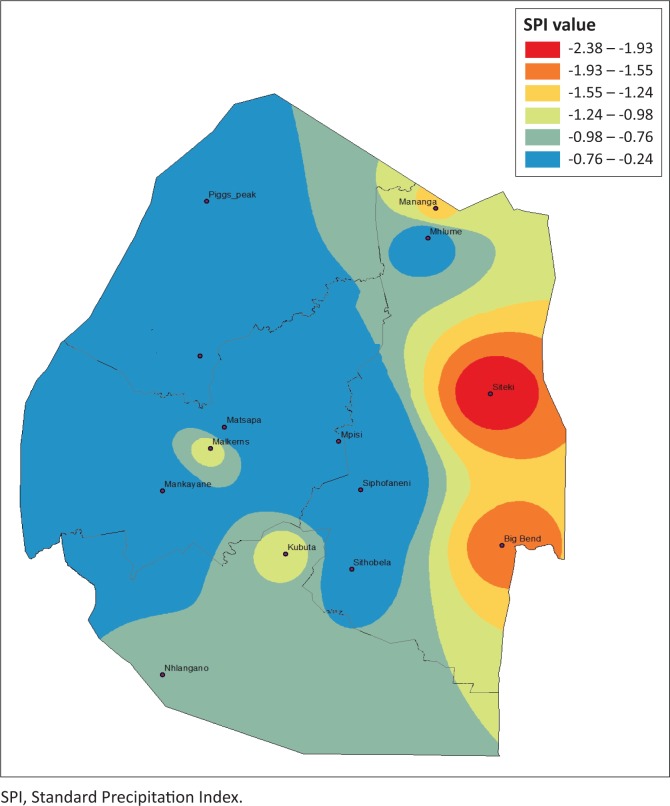
Standard Precipitation Index 3-month timescale during 1985–1986.

**FIGURE 7:**
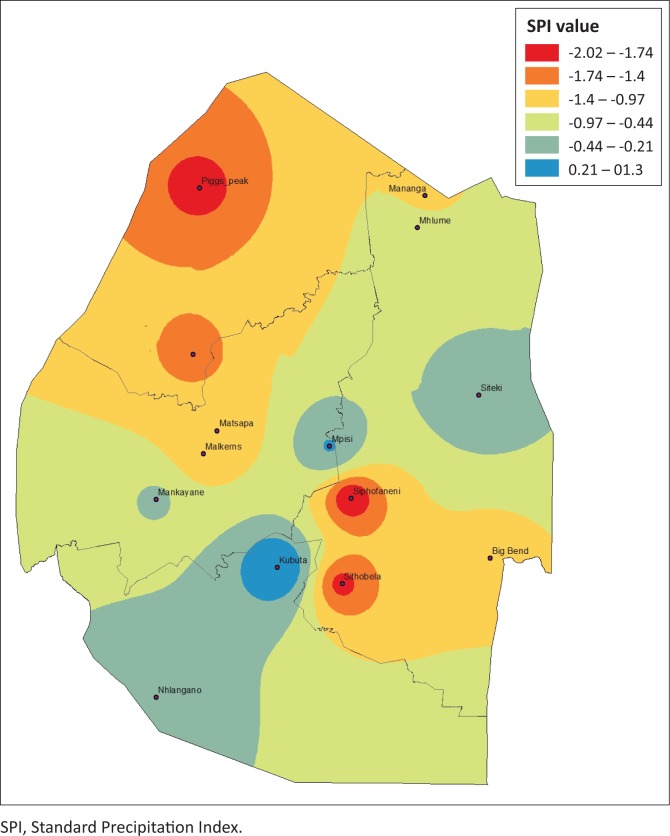
Standard Precipitation Index 3-month timescale during 2004–2005.

**FIGURE 8:**
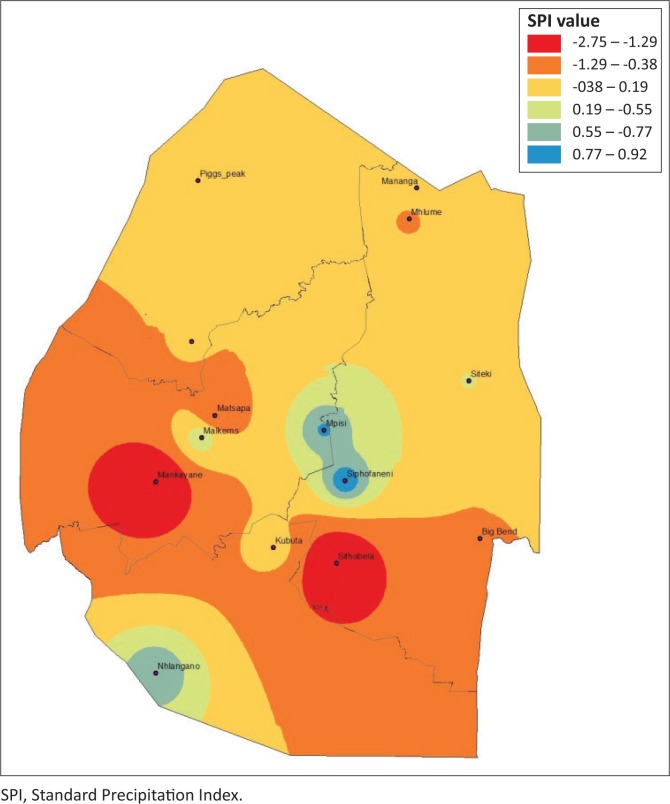
Standard Precipitation Index 3-month timescale during 2005–2006.

**FIGURE 9:**
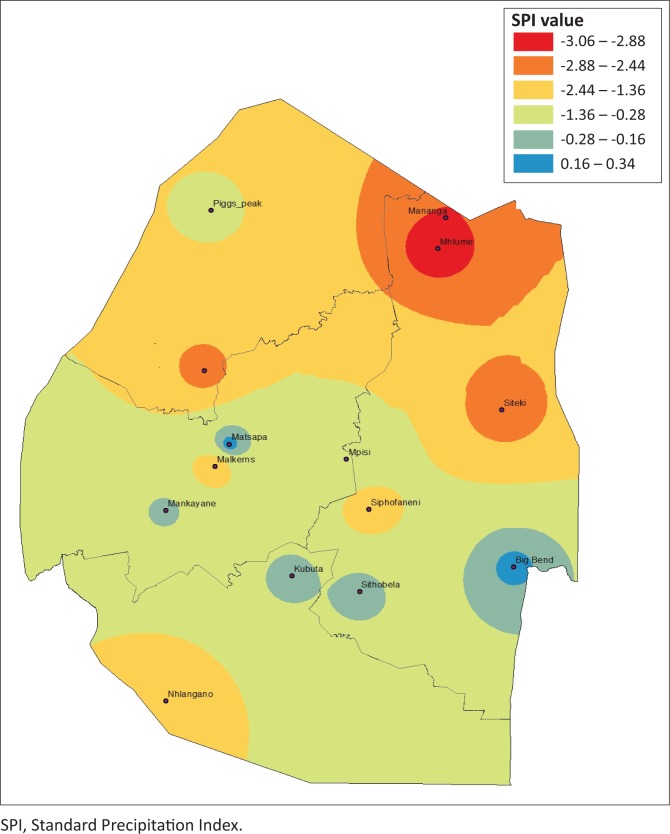
Standard Precipitation Index 3-month timescale during 2015–2016.

Estimating agriculture drought severity at a station or AEZ provides useful information for drought planning and management. It is therefore important to assess the drought over a specified agro-ecological region. This allows the administrative areas that fall within these regions to plan effectively. The drought analysis based on these zones is useful for determining the spatial distribution and characteristics of drought and for evaluating the most affected areas for a specific drought event. To provide a complete picture of the drought hotpots in Eswatini, spatial analysis was performed by plotting 3-month SPI values using ArcGIS 10.1. Figures 6–9 depict the spatial extent of selected drought years in Eswatini from 1986 to 2016. All the interpolated SPI maps were reclassified into four classes, that is, SPI value from −1 to 1 as no drought, −1.5 to −1.0 as moderate drought, ≤ 2 to −1.5 as severe drought and ≥ 2 as extreme drought category.

In 1986, moderate and extreme droughts were mostly experienced in the Lowveld AEZ, whereas between 2004 and 2006 moderate to extreme droughts were experienced countrywide. The results identified that there were spatial differences in moderate, severe, extreme and no drought events experienced in the study period. The 2005–2006 and the 2015–2016 droughts were most severe and can be observed by spatial coverage indicated in red. The 1986 drought was less severe, with less spatial coverage (in red). Moderate and extreme droughts were mostly experienced in the Lowveld AEZ, whereas between 2004–2005 and 2005–2006 moderate to extreme droughts were experienced countrywide. There were significant differences in the analysis of droughts across administrative areas and AEZs. Field surveys and information sourced from key informants have verified that these droughts were the most severe, with the greatest impact being felt in the Lowveld and Middleveld. However, the impact of the 2015–2016 drought was felt countrywide with massive crop and livestock losses. Therefore, estimating drought severity at a station or AEZ provides useful information for drought planning and management. Understanding the spatial extent allows effective planning within the administrative areas that fall within these regions. The spatial differences across the country therefore allow for localised drought monitoring, enabling more accurate area-specific results, and therefore area-specific drought management planning.

## Conclusion and recommendation

The climate of the African continent and Eswatini in particular exhibits large geospatial and temporal variability. This study was focused on presenting the analysis of the temporal and spatial characteristics of droughts in Eswatini. The 3-, 6- and 12-month SPIs were computed to determine drought severity and onset of meteorological drought in the country. Overall, the SPI effectively described the drought conditions in Eswatini. The analysis of droughts during 1986–2017 indicated that droughts have intensified in terms of their frequency, severity and geospatial coverage over the last few decades. The results demonstrated that moderate droughts are most prevalent in Eswatini, while the frequency of severe and very severe droughts is low. Most parts of the country were vulnerable to mild and moderate agricultural drought (3 and 6 months) timescales. The worst drought years were the 1985–1986, 2005–2006 and 2015–2016 agricultural seasons, and this was evident from the SPI at 3-, 6- and 12-month timescales. The drought periods were consistent with drought declarations by the government as well as the EM-DAT database.

At national level and agro-ecological level, severe droughts were experienced over the last few decades, for instance, in 1990, 2001, 2004, 2006 and, recently, in 2016. There were temporal and spatial differences across the country as well as AEZs. The spatial SPI visualisation has the ability to provide drought management planners a tool for immediate drought categorisation. The drought analysis (using the SPI at different timescales) affirms the applicability of the SPI for drought monitoring in Eswatini. The spatiotemporal drought analysis can provide information for early warning, particularly in drought-prone areas, by depicting a drought before the effects are felt. Because of the differences in drought severity at different timescales, it is recommended that the SPI should be used for drought monitoring in combination with other indices and approaches such as vulnerability assessments and remote sensing.
